# Off-Label Medicine Use in Pediatric Inpatients: A Prospective Observational Study at a Tertiary Care Hospital in India

**DOI:** 10.1155/2014/415815

**Published:** 2014-11-25

**Authors:** Mohd Masnoon Saiyed, Tarachand Lalwani, Devang Rana

**Affiliations:** ^1^Department of Pharmacology and Clinical Pharmacy, K.B. Institute of Pharmaceutical Education and Research, GH 6, Sector 23, Gandhinagar, Gujarat 382024, India; ^2^Department of Pharmacology, Smt. N.H.L. Municipal Medical College, Sheth V.S. General Hospital, Ellisbridge, Ahmedabad, Gujarat 380006, India

## Abstract

*Background*. In the absence of standard pediatric prescribing information, clinicians often use medicines in an off-label way. Many studies have been published across the globe reporting different rates of off-label use. There is currently no study based on Indian drug formulary. *Methods*. The prospective observational study included pediatric patients in ages between 0 and 12 years admitted in a tertiary care hospital. Off-label use was assessed using the National Formulary of India (NFI). Predictors of off-label use were determined by logistic regression. *Results*. Of the 1645 medications prescribed, 1152 (70%) were off-label based on 14 possible off-label categories. Off-label medicines were mainly due to dose difference and use in restricted age limits as indicated in NFI. Respiratory medicines (82%), anti-infectives (73%), and nervous system medicines (53%) had higher off-label use. Important predictors of off-label prescribing were pediatric patients in age of 0 to 2 years (OR 1.68, 95% CI; *P* < 0.001) and hospital stay of six to 10 days (OR 1.91, 95% CI; *P* < 0.001). *Conclusion*. Off-label prescribing is common among pediatric patients. There is need to generate more quality data on the safety and efficacy of off-label medicines to rationalize pediatric pharmacotherapy.

## 1. Introduction 

The goal to achieve ideal pediatric drug therapy is a worldwide challenge for regulatory bodies and clinicians. Children are treated with medicines not tested for safety and efficacy and are frequently supported by the low quality of evidence [1]. In the absence of standard prescribing information, clinicians prescribe drugs in an off-label manner. Off-label use means use of medicine which is outside the terms of product license with respect to dose, route of administration, indication, or age [2]. Off-label medicine use among children represents an important health issue as the effects and health risks may be unexpected. Various national and international studies have been published about the amount of and problems associated with off-label medicines in children. The magnitude of off-label prescribing is accounted to be between 18% and 60% in infants but it may be up to 90% in neonates [3–9].

Most of these studies have been conducted outside India and may not be applicable as hospitalization and prescribing patterns differ based on culture, healthcare infrastructure, and health policies. There is a distinct lack of research on off-label prescribing in India. Only one previous study has been conducted in India which reported 50.62% of off-label prescribing based on British National Formulary [5]. Currently there is no study conducted in India based on National Formulary of India (NFI). Hence, the objective of the research study was to quantify off-label use based on NFI, various predictors and discusses some strategies to monitor it.

## 2. Methods

### 2.1. Data Collection

The prospective observational study was carried out at a tertiary care hospital in Ahmedabad (India) for the period of six months. All the pediatric patients in ages between 0 and 12 years receiving at least one medication and admitted in pediatric ward were included in the study. The pediatric ward was of 60 bed size and attended by 10 academic pediatricians and 28 postgraduate students. Patients were not considered if they were in age of more than 12 years, not taking any prescription medication and undergoing surgery. Demographics, clinical characteristics, and medication usage were obtained from the medical records using predefined paper case record forms. The data collection was carried out by one of the researchers (MMS) who discussed each of the medicines with attending pediatrician and clinical pharmacologist (DR). Drugs were entered into the database using the World Health Organization Anatomical Therapeutic Chemical (WHO-ATC) classification system [11]. We utilized National Formulary of India (NFI, 4th edition, 2011) which is an official publication of Indian Pharmacopoeia Commission, Ministry of Health and Family Welfare, Government of India [10]. There were five off-label categories (Table 1). Categories for off-label use were allocated for each medicine according to the reason(s) why their use was deemed off-label when compared to the terms of the product labeling for that medicine in NFI. We followed a published algorithm and used these dimensions to determine whether there is strong scientific evidence for frequently prescribed off-label medicines [12] (Table 4).

### 2.2. Statistical Analysis

Variables such as age, number of medications, number of diagnoses, and length of hospital stay were regarded as continuous and expressed as mean with standard deviation (SD). Categorical variables are presented as numbers with percentages (%). Risk factors for off-label prescribing were analyzed with multivariate binary logistic regression. The odds ratio (OR) with 95% confidence interval (CI) was used to determine the predictors for off-label prescribing. A probability value of less than 0.05 was considered statistically significant for all analyses. The data were analyzed using Statistical Package for the Social Sciences (SPSS Inc., IBM).

### 2.3. Research Reporting

This study was reported according to the Strengthening the Reporting of Observational Studies in Epidemiology (STROBE) guidelines [13].

## 3. Results

### 3.1. Demographic and Clinical Characteristics

The study included a total of 320 patients admitted in pediatric general ward of the public teaching hospital over a period of six months. There were 206 (64%) male and 114 (36%) female patients. The mean age was 2.73 years (range = 0.1 to 12 years and standard deviation: 3.09). The most common age group was 0 to 1 years representing 43% of total sample size.

On average each patient had 1.2 number of diagnosis and 48 patients (mean = 2.5) had more than one diagnosis during hospitalization. The majority of patients suffered from respiratory diseases (33%), central nervous system diseases (16.5%), and gastrointestinal disease (11%). A total of 1645 medications were prescribed to the study cohort during hospitalization, with a total hospital stay of 1743 days. This constituted the mean number of five (SD = 2) medications prescribed per patient and 5.48 (SD = 3.62) days of hospital stay. The number of medications and hospital stay ranged from one to 13 and one to 33 days, respectively. The majority of patients received antibiotics, cough, and cold preparations, antipyretics, inhaled corticosteroids, bronchodilators, and antiepileptic drugs. Medications were mostly administered by oral route (40%) and intravenous route (35%) followed by inhalation route (25%).

### 3.2. Nature of Off-Label Prescribing

Of the 320 patients included in the study, 310 (97%) patients received at least one off-label medication. A total of 1645 medications were administered during the hospitalization; 1152 (70%) medicines were prescribed in off-label manner when its usage was validated with National Formulary of India, 2011, for five different off-label categories. Patients received on average (SD) 3.66 (2.13) off-label medicines within a range of one to 10. The most common cause of off-label prescribing 893 (63%) was due to higher dose use (category 1) than stated for particular pediatric patients. Mostly ceftriaxone, amikacin, paracetamol, and chlorpheniramine were prescribed in this category.

Off-label medicines use for age (category 2) and indication (category 3) was found to be 282 (19.8%) and 145 (10.2%), respectively. Those medicines which had no pediatric information (category 5) were 76 (5.3%). Use of ipratropium, salbutamol, and dextromethorphan was common for different dose and age limits. Lorazepam (seizures) and ondansetron (nausea and vomiting) were frequently used for off-label indication.

### 3.3. Off-Label Prescribing in Different Age Groups

The study also attempted to distribute the receipt of off-label medicines in different age groups as shown in Table 2. The extent off-label prescribing was highest (76%) in age group of more than 1 to 2 years, followed by age group of more than 1 to 12 months which accounted for 69% of off-label use. Off-label use in dose (category 1) was consistent among the patients in age of 0 to 6 years. The use of medicines in patients for restricted age limits (category 2) was highest in 1–12-month age group. Indication (category 3) and absence of pediatric information (category 5) were most prominent in 6–12-year patients.

### 3.4. Off-Label Prescribing by ATC Class and Drugs

Highest proportion of off-label medicines were prescribed in anti-infectives for systemic use 389 (73%) and respiratory system 378 (82%) as shown in Table 3. The amount of off-label prescribing in nervous system and alimentary tract and metabolism system was 220 (53%) and 85 (86%), respectively. Majority of off-label medicines prescribed were ceftriaxone, amikacin, amoxicillin, and vancomycin in antibiotics class and inhaled corticosteroids, ipratropium, salbutamol, chlorpheniramine, phenylephrine corresponding to respiratory system. Blood and blood forming agents 16 (89%), hormonal preparation 32 (80%), and cardiovascular medicines 24 (71%) had substantial high amount of off-label use. Adrenaline, nifedipine, prednisolone, and dexamethasone were also used in off-label manner. Antiparasitic products 8 (16%) had minimum off-label use.

Antibiotics (75%) and inhaled corticosteroids (79%) were mostly prescribed in higher doses (category 1) than recommended. In respiratory system, many medicines (39.5%) were prescribed below age limits (category 2) as indicated in the formulary. Medicines used for unapproved indication (category 3) largely confined to alimentary tract and metabolism system and blood and blood forming agents. Medicines which had no pediatric information were predominantly in antihypertensive drug class.

### 3.5. Predictors of Off-Label Medicine Use

On multivariate regression analysis as shown in Table 5, we found that pediatric patients in age of 0 to 2 years (OR 1.68, 95% CI, 1.26–2.24,) were more likely to receive off-label medicines than any other age group. Female patients (OR 1.41, 95% CI, 1.13–1.77) received substantially high amount of off-label medicines compared to male. Hospital stay of six to 10 days (OR 1.91, 95% CI, 1.33–2.75) also carried higher risk of off-label prescription.

## 4. Discussion

Using the data of the pediatric ward of a tertiary care hospital, we found that 70% of medicines were prescribed in off-label manner. The magnitude off-label prescribing is substantial higher than reported in the recent studies [14–16]. Cuzzolin et al. performed a literature review of published studies on off-label and unlicensed drug use in children [3]. A total of 30 studies from 1990 to 2006 were included. Seven studies involved neonatal intensive care units (NICUs), fifteen studies included pediatric wards, and twelve studies were conducted in community setting. Cuzzolin et al. found off-label prescribing in pediatric ward ranging between 18 and 60%. Various reasons for different rates of off-label use are off-label classification methods, sample sizes, pediatrician's prescribing habits, in-house treatment protocols, and diseases characteristics most importantly pediatric drug regulation.

Patients in ages between 0 and 2 years are most likely to receive off-label medicines and had highest representation in study sample. Previous studies also established that age group of 0 to 2 years is the highest recipient of off-label prescriptions [16, 17]. This is mainly due to absence of specific dosing guidelines and route administration in 0-to-2-year age group in NFI. Of the medicines prescribed during the period, it was observed that antibiotics, respiratory medicines, and nervous system medicines were frequently prescribed in off-label manner. Several studies had shown high rate of off-label prescribing in respiratory [18], antibiotics [19], analgesics [20], and antiepileptics [21]. About 82% of medicines in respiratory system were off-label which is more than what is reported in studies in US (*n* = 312 million, 70%) [22] and Portugal (*n* = 500, 77%) [23]. If asthma therapies are considered, inhaled corticosteroids are frequently prescribed (30.7% of all prescriptions). The mainstay therapy for asthma is inhaled corticosteroids (ICS), but guidelines often do not give specific recommendations for upper doses limits specially in children [24].

Various combinations of antihistaminics, decongestants, and/or analgesics were prescribed to patients in off-label doses for common cold. But the effectiveness in young children is still questionable [25]. During the study paracetamol was commonly used drug to treat fever and pain in children. Still, the off-label use of paracetamol is substantial, mainly due to off-label classification for dose or age. Although paracetamol is normally considered safe in pediatrics care, a previous Cochrane review pointed out that there is limited evidence regarding the efficacy and safety for paracetamol in the treatment of fever in children [26]. Lorazepam and ondansetron were widely prescribed for off-label indication but are supported by well conducted clinical studies [27, 28].

World Health Organization (WHO) adopted in 2007 the WHA60.20 Resolution “Better Medicines for Children” to improve monitoring medicine safety in the pediatrics and highlighted its concern on off-label medicines [29]. The strict drug approval procedure is the way to ensure quality data on quality, safety, and efficacy for different pediatric age groups. Despite many regulatory amendments and policies, we still have apathetic outlook to pediatric clinical trials [30].

The pharmaceutical companies should be convinced to have appropriate pediatric information in the label and they are not being permitted to market drugs likely to be used in children without suitable pediatric labeling. Indian drug regulatory authorities should also develop pediatric specific drug development regulation so that the tendency to market medicines without pediatric specific data is discouraged. This might ensure pediatric labeling for new medicines yet to be introduced in the market; but it is unlikely that drug companies will carry out trials to confirm pediatric use for drugs already marketed and used in children, although in an off-label manner.

Alternatively, the situation can be improved when prescribers report their pediatric experiences with different off-label medicines in form research articles or discussion at scientific platforms. When medicines are used as off-label, each patient is unique and risk-benefit pertaining to him should be assessed by high quality evidence. The doctor needs to be updated with latest evidence which could be accomplished by using several useful drug compendia like DRUGDEX, Clinical Pharmacology, and so forth. Only such focused and coordinated actions would make sure that children's right to safe, cost-effective, and quality medicines would be realized.

## 5. Conclusion

Based on National Formulary of India, our data suggest that magnitude of off-label prescribing in pediatric inpatients is considerable higher than reported in some of the countries. Dose discrepancy and use in restricted age limits were identified as main contributor to off-label prescribing. There is need for strict drug regulation for pediatric population to ensure safety and effectiveness of pharmacotherapy. Further studies are needed to examine why there are inadequate dosing guidelines and generation of more clinical data especially in respiratory medicines. Understanding various risk factors and spectrum of off-label medicine use can assist developing prevention strategies. Off-label prescribing is a reality and will not go soon. Implementing evidence based approach can significantly improve rationality of pediatric pharmacotherapy.

## Figures and Tables

**Table 1:** Off-label medicine use categories.

Sr. number	Category	Reason for off-label use	Examples based on NFI
1	Dose	Dose higher than recommended	Once daily dosing of gentamicin in children
2	Age	Drug not recommended in the patient below a certain age	Losartan used in children under 6 years
3	Indication	Drug prescribed for indications outside of those listed in the NFI	Diclofenac for abdominal pain
4	Route of administration	Drug administered by a route not described in the NFI	Adrenaline through inhalation route
5	Absence of pediatric information (PI)	No mention at all in the NFI regarding pediatric use	Nifedipine for hypertension

^*^NFI: National Formulary of India, 2011 [10].

**Table 2:** Proportion off-label medicine use in different age groups.

Age group	Total medicine	Off-label medicine	Off-label category				
			Dose	Age	Indication	Route	Absence of pediatric information
All age groups (*n* = 320)	1645	1152 (70)	893 (63)	282 (19.8)	145 (10.2)	24 (1.7)	76 (5.3)

1–12 months (*n* = 138)	683	472 (69)	391 (64)	147 (24)	44 (7.2)	8 (1.4)	21 (3.4)

>1-2 years (*n* = 63)	363	276 (76)	218 (63)	70 (20.3)	35 (10.3)	6 (1.8)	16 (4.6)

>2 years–6 years (*n* = 74)	393	269 (68)	204 (65.4)	46 (14.7)	37 (11.8)	5 (1.6)	20 (6.5)

>6–12 years (*n* = 45)	206	135 (65)	80 (52.6)	19 (12.5)	29 (19.1)	5 (3.3)	19 (12.5)

^*^Note: the total number of off-label uses exceeds that of off-label drugs because a drug may be off-label for more than one category. Figures in parentheses indicate percentage.

**Table 3:** Off-label medicine use according to ATC and drugs.

WHO-ATC system	Total medicine	Off-label medicine	Off-label category				
			Dose	Age	Indication	Route	Absence of pediatric information
Anti-infectives for systemic use	531	389 (73)	408 (87.8)	21 (4.5)	5 (1.1)	9 (1.9)	22 (4.7)

Respiratory system	463	378 (82)	244 (56.8)	170 (39.5)	6 (1.4)	7 (1.6)	3 (0.7)

Nervous system	411	220 (53)	168 (55.8)	49 (16.3)	68 (22.6)	0 (0)	16 (5.3)

Alimentary tract and metabolism	99	85 (86)	34 (27.7)	18 (14.6)	48 (39)	0 (0)	23 (18.7)

Hormonal preparation	40	32 (80)	30 (66.7)	15 (33.3)	0 (0)	0 (0)	0 (0)

Cardiovascular system	34	24 (71)	9 (28.1)	9 (28.1)	2 (6.3)	0 (0)	12 (37.5)

Blood and blood forming agents	18	16 (89)	0 (0)	0 (0)	16 (100)	0 (0)	0 (0)

Ant parasitic products	49	8 (16)	0 (0)	0 (0)	0 (0)	8 (100)	0 (0)

^*^Note: the total number of off-label uses may exceed that of off-label drugs because a drug may be off-label for more than one category. Figures in parentheses indicate percentage.

**Table 4:** Most frequently prescribed off-label medicines.

Sr. number	System	Off-label medicine use (%)	Off-label category	Quality of evidence	Strength of recommendation
1	Alimentary tract and metabolism	Ondansetron (100%)	Indication	Strong	High
2	Respiratory system	Phenylephrine (91%)	Age, dose	Moderate	Medium
3	Respiratory system	Dextromethorphan (90%)	Age, dose	Moderate	Medium
4	Respiratory system	Chlorpheniramine (88%)	Dose	Moderate	Medium
5	Anti-infectives for systemic use	Ceftriaxone (81%)	Dose	Strong	High
6	Nervous system	Lorazepam (80%)	Indication	Strong	High
7	Respiratory system	Ipratropium (79%)	Age, Dose	Strong	High
8	Anti-infectives for systemic use	Amikacin (75%)	Dose	Strong	High
9	Respiratory system	Salbutamol (75%)	Age, dose	Strong	High
10	Nervous system	Paracetamol (56%)	Age, dose	Moderate	High

**Table 5:** Predictors of off-label medicine use (multivariate binary logistic regression model).

Sr. number	Parameter	Total number of patients	OR (95% CI)	*P* value
1	Age group			
	0–2 years	201	1.68 (1.26–2.24)	<0.001
	>2–6 years	74	1.40 (0.99–1.96)	0.052
	>6–12 years	45	1 (reference)	—

2	Gender			
	Female	114	1.41 (1.13–1.77)	0.002
	Male	206	1 (reference)	—

3	Number of diagnosis			
	Double or more	48	0.91 (0.73–1.12)	0.389
	Single	272	1 (reference)	—

4	Hospital stay			
	1–5	224	1.32 (0.97–1.81)	0.074
	6–10	66	1.91 (1.33–2.75)	<0.001
	More than 10	30	1 (reference)	—

^*^ *P* < 0.05 indicates significant difference and *P* > or = 0.05 indicates nonsignificant difference; OR: odd ratio and CI: confidence interval.
